# Leisure Activities, *APOE* ε4, and Cognitive Decline: A Longitudinal Cohort Study

**DOI:** 10.3389/fnagi.2021.736201

**Published:** 2021-09-20

**Authors:** Yun Zhang, Shihui Fu, Ding Ding, Michael W. Lutz, Yi Zeng, Yao Yao

**Affiliations:** ^1^School of Sociology and Anthropology, Sun Yat-sen University, Guangzhou, China; ^2^Department of Cardiology, Hainan Hospital of Chinese People's Liberation Army General Hospital, Sanya, China; ^3^Institute of Neurology, Huashan Hospital, Fudan University, Shanghai, China; ^4^Department of Neurology, Duke University School of Medicine, Durham, NC, United States; ^5^Center for Healthy Aging and Development Studies at National School of Development, Peking University, Beijing, China; ^6^Center for the Study of Aging and Human Development and Geriatrics Division, Medical School of Duke University, Durham, NC, United States; ^7^China Center for Health Development Studies, Peking University, Beijing, China

**Keywords:** *APOE*, leisure activity, cognitive decline, older adults, cohort study, CLHLS

## Abstract

**Background:** Both leisure activities and the ε4 allele of the apolipoprotein E (*APOE* ε4) have been shown to affect cognitive health. We aimed to determine whether engagement in leisure activities protects against *APOE* ε4-related cognitive decline.

**Methods:** We used the cohort data from the Chinese Longitudinal Healthy Longevity Survey. A total of 3,017 participants (mean age of 77.0 years, SD = 9.0; 49.3% female) from 23 provinces of China were recruited in 2008 and were reinterviewed in 2014. We assessed cognitive function using the Mini-Mental State Examination (MMSE). We calculated cognitive decline using subtraction of the MMSE score of each participant in 2008 and 2014. We genotyped a number of *APOE* ε4 alleles for each participant at baseline and determined the Index of Leisure Activities (ILAs) by summing up the frequency of nine types of typical activities in productive, social, and physical domains. We used ordinal logistic regression models to estimate the effects of leisure activities, *APOE* ε4, and their interaction on cognitive decline, statistically adjusted for a range of potential confounders.

**Results:** There were significant associations between *APOE* ε4 and faster cognitive decline, independent of potential confounders, and between leisure activities and mitigated cognitive decline. The odds ratios were 1.25 (95% CI: 1.03, 1.53) and 0.93 (95% CI: 0.89, 0.97), respectively. We found significant interactions of *APOE* ε4 with leisure activities with a *P*-value of 0.018. We also observed interactive effects of subtypes of leisure activities: participants who regularly engaged in productive activities were more likely to reduce the risk of *APOE* ε4-related cognitive decline.

**Conclusion:** Our findings provide support for the indication that participating in leisure activities reduces the risk of *APOE* ε4-related cognitive decline.

## Highlights

- Engagement in leisure activities is associated with a lower risk of cognitive decline, while carriage of an *APOE* ε4 allele is associated with faster cognitive decline.
- Leisure activities reduce the risk of *APOE* ε4-related cognitive decline.
- The modification effect seems more marked in productive and social activities, while less significant in physical activities (PAs).

## Introduction

With rapid population aging worldwide, cognitive disorders, such as dementia, pose a serious concern for public health and to the social and economic development of modern society (Decarli, 2003; Fox and Petersen, 2013). Dementia is a major cause of disability and for the institutionalization of older people. There is no effective disease-modifying treatment currently available (Norton et al., 2013; Livingston et al., 2020). A substantial amount of evidence shows that both genetic and lifestyle factors play a role in determining the individual risk of dementia and cognitive impairment. The ε4 allele of the apolipoprotein E (*APOE* ε4) gene is the strongest known genetic risk factor for dementia and cognitive impairment, and it may represent roughly half of the genetic risk for neuropathologically based cognitive diseases (Raber et al., 2004; Caselli et al., 2010; Lim et al., 2018). Nevertheless, the genetic risk of *APOE* ε4-related cognitive decline could be partly offset by favorably modifiable factors, such as higher education, more intake of protein-enriched food and choline, and maintaining vascular health (Ferrari et al., 2013; Sienski et al., 2021; Zhang et al., 2021). More specifically, established studies have suggested the protective effects of these modifiable factors, including engaging in leisure activities, were more pronounced in *APOE* ε4 carriers (Goodwin et al., 1983; Ott et al., 1998; Huang et al., 2005; Rovio et al., 2005; Kivipelto et al., 2008).

Leisure activities have generally been thought to improve cognitive health, per the “use it or lose it” hypothesis (Salthouse, 2006). It should be acknowledged that there are varied types of leisure activities, typically physical, social, and productive activities. The three leisure activities have varied pathways which converge within three major etiological hypotheses for dementia and other cognitive disorders: the cognitive reserve hypothesis, the vascular hypothesis, and the stress hypothesis (Fratiglioni et al., 2004). It is valuable to differentiate types of leisure activities when studying their effects on cognitive decline, especially for the people who carry an *APOE* ε4 allele, since it would provide useful information for designing effective intervention programs to prevent cognitive decline and other neurodegenerative diseases. In addition, the extant two studies with either relatively small sample size or short duration of follow-up reported inconsistent results, regarding the interaction effect of *APOE* ε4 with physical, social, and productive activities on cognitive health (Niti et al., 2008; Yang et al., 2015). We, therefore, used a nationwide, community-based longitudinal dataset with a relatively long duration of follow-up (about 6 years) to test the associations of *APOE* ε4 allele carriage and engagement in leisure activities with cognitive decline and to investigate whether there were interactive effects of *APOE* ε4 carriage with leisure activities. We also evaluated associations of the three leisure activity subtypes on cognitive decline. We hypothesized that there would be a significant statistical interaction where carriers of the *APOE* ε4 allele who engaged in leisure activities would have lower odds of cognitive decline compared to the noncarriers.

## Methods

### Study Design and Participants

We used data from the 2008 wave of the Chinese Longitudinal Healthy Longevity Study (CLHLS). The CLHLS applied a multistage, stratified cluster sampling design in 23 of 31 provinces in China. With 631 cities and counties randomly selected as the sample sites, the study sample roughly represents about 85% of the Chinese population. Details of CLHLS have been reported elsewhere, and extensive evaluations of the data quality of the CLHLS have shown that the data from the CLHLS surveys are of good quality for research (Zeng et al., 2016).

Among the 7,972 participants enrolled in the 2008 wave of CLHLS with *APOE* genotyping data available, 3,106 died during the follow-ups conducted in 2014, and 1,554 were lost to follow-up or refused the reinterview. In addition, we excluded those who had severe cognitive impairment [Mini-Mental State Examination (MMSE) score < 18] at baseline in the 2008 survey to rule out recall bias regarding the engagement in leisure activities and key covariates (An and Liu, 2016). Therefore, a total number of 3,312 participants were re-interviewed in 2014. Among them, 267 were excluded due to missing data of MMSE, leisure activities, and other covariates; and 28 participants were excluded as outliers in the trajectory of cognitive decline (those with a difference in participant MMSE scores in 2008 and 2014 that was >5). A total of 3,017 participants were included in the analysis of our study (Supplementary Figure 1: Study profile).

Chinese Longitudinal Healthy Longevity Study was approved by the Biomedical Ethics Committee, Peking University (IRB00001052–13074). All participants or their legal representatives provided written, informed consent for every interview. This study followed the Strengthening the Reporting of Observational Studies in Epidemiology (STROBE) reporting guidelines.

### Cognitive Assessment

Each participant in the study was administered the MMSE in the 2008 baseline survey and the follow-up survey of 2014. As a screening tool for dementia and cognitive impairment, the MMSE is frequently used to track changes in cognitive function over time. It is recognized as a reliable tool to detect cognitive impairment, especially among those who are not able to go through complex clinical diagnostic testing (Pezzotti et al., 2008). MMSE scores range from 1 to 30, with a higher score indicating better cognitive function (Cummings, 1993).

We calculated cognitive decline by subtracting the MMSE score of each participant in 2014 from the score in 2008. Consistent with previous studies, those who had an MMSE score drop of 5 or more points during the period were defined as showing substantial decline (clinically meaningful deterioration), while those who have decreased 1–4 points of MMSE score during the period are defined as moderate decline, and those whose MMSE scores were not decreased, along with those whose MMSE scores were increased, during the period are defined as nondecline (Doody et al., 2001; Zhang et al., 2021).

### Leisure Activities Assessment

In this study, we considered nine typical leisure activity components, which were taken at present such as housework, reading newspapers/books, watching tv/listening to the radio, playing cards/mah-jongg, engagement in social work, traveling, doing fieldwork, practicing labor work, and exercising. These components were reported at baseline. We then grouped the nine leisure components into three categories: productive activity (housework, reading newspapers/books, and watching tv/listening to the radio), social activity (playing cards/mah-jongg, engagement in social work, and traveling), and physical activity (PA) (doing fieldwork, practicing labor activity, and exercising), in accordance with established studies (Niti et al., 2008; Yang et al., 2015). The participants were asked to report their frequency of participating in each activity. For each leisure component, other than traveling, the frequency was categorized into three scores: “almost every day,” (scored as 2 points) “sometimes or occasionally,” (scored as 1 point) and “rarely or never” (scored as 0 points). The frequency of travel was categorized into “more than twice/year” (scored as 2 points), “once/year” (scored as 1 point), and “rarely or never” (scored as 0 points). Therefore, the sum of scores of productive activities, social activities, and PAs ranged from 0 to 6. We determined the Index of Leisure Activities (ILA) by summing up the scores of nine leisure components for each participant. The actual range of ILA was from 0 to 17, with a theoretical range of 0 to 18.

### APOE Genotyping

The DNA samples of the participants in this study were genotyped by Beijing Genomics Institute using a well-designed customized chip (Illumina HumanOmniZhongHua-8 BeadChips) targeting ~27,000 longevity-related single nucleotide polymorphisms (SNPs). These candidate SNPs were selected based on previously published associations with longevity, chronic diseases, and health indicators. Further details on the genotyping platform, sample filtering, and quality control can be found in our published work (Zeng et al., 2016; Liu et al., 2021). Consistent with prior studies, we determined APOE genotypes using the two SNPs: rs429358 and rs7412 (Zhu et al., 2020). The two APOE ε SNPs were both in Hardy-Weinberg equilibrium (*P* > 0.05) assessed with PLINK 1.90 beta. There were six APOE genotypes observed in our participants, namely ε2/ε2, ε2/ε3, ε2/ε4, ε3/ε3, ε3/ε4, and ε4/ε4. We dichotomized the participants into *APOE* ε4 noncarriers (including ε2/ε2, ε2/ε3, and ε3/ε3), and ε4 carriers (including ε2/ε4, ε3/ε4, and ε4/ε4).

### Covariates

The potential confounders of demographics, socioeconomic status, health status, and lifestyles were addressed since established studies have suggested they were significantly associated with cognitive decline. Therefore, in the current study, we assessed a range of these potential confounders by including the covariates age, sex, years of education, occupation before retirement, marital status, self-rated socio-economic status, body mass index (BMI), alcohol drinking, smoking, diversity of intaking protein-enriched foods, and four clinician-diagnosed chronic conditions from self-reports: hypertension, diabetes, heart disease, and cerebrovascular disease.

We calculated age based on interview dates and self-reported birth dates, which were verified by family members, genealogical records, ID cards, and household registrations. We dichotomized marital status to married and not married at the time of the interview. We defined the main occupation before 60 years of age as white collar, blue collar, and others. Self-reported socio-economic status was categorized into three levels (favorable, intermediate, and unfavorable). Marital status referred to living with a married spouse or having no spouse. Height was measured to the nearest 1 cm in the 2008 survey or was estimated based on knee height (vertical distance from the sole of the foot to the upper surface of the knee, with the knee and ankle each flexed to a 90° angle) using a validated equation in the CLHLS (men, height = 67.78 + 2.01 × knee height; women, height = 74.08 + 1.81 × knee height) (Zhang et al., 1998). BMI was calculated as the weight of a person in kilograms, divided by height in meters squared. The participants provided their frequency of intake of six protein-enriched foods (meats, fish, eggs, nuts, bean products, and dairy products), which was categorized into three groups: “almost every day,” (scored as 2 points) “sometimes or occasionally,” (scored as 1 point) or “rarely or never” (scored as 0 points). We calculated the diversity of dietary protein intake (DDPI) by summing up the scores of intake frequency of the six protein-rich foods (Zhang et al., 2021). Smoking at present, alcohol drinking at present, and the four clinician-diagnosed chronic conditions were dichotomized as yes vs. no.

### Statistical Analysis

Data were described using mean and SD (continuous variables with normal distribution), median and interquartile range (continuous variables with skewed distribution), and number and percentage (categorical variables). Data were compared between participants by levels of cognitive decline (substantial decline, moderate decline, and nondecline), we used the one-way ANOVA for continuous data and Chi-square for categorical data. Ordinal logistic regressions were used to analyze the associations between *APOE* ε4 (carrier vs. noncarrier), ILA, three subtypes of leisure activities, and the degree (ordered three strata: none, moderate, and substantial) of cognitive decline. We investigated the independent effects of the carriage of *APOE* ε4 allele, ILAs, and each subtype of leisure activities on cognitive decline in four models (Model 1, unadjusted model; Model 2, adjusted for sex, age, and BMI; Model 3, further adjusted for education, occupation before retirement, marital status, smoking, alcohol drinking, and DDPI, based on Model 2; and Model 4, further adjusted for hypertension, diabetes, heart disease, and cerebrovascular disease based on Model 3). We report descriptive statistics according to EQUATOR guidelines for health research. We also included the breakdown of e4 carriers into three groups: *APOE* ε2 (ε2/ε2 and ε2/ε3), ε3 (ε3/ε3), and ε4 (ε3/ε4 and ε4/ε4) to examine the association of carrying *APOE* ε2 allele and cognitive decline in our study.

To analyze the 2-way gene-environment (GxE) interactions of *APOE* ε4 and leisure activities on cognitive decline, we dichotomized the leisure activities and its components and then added interaction terms of *APOE* ε4 genotype with dichotomous mean values of leisure activities (≥8 vs. ≤ 7), productive activities (≥3 vs. ≤ 2), social activities (≥1 vs. =0), and PAs (≥3 vs. ≤ 2) in the fully adjusted ordinal logistic regression model (Model 4), respectively. We also performed a three-way interaction analysis of *APOE* ε4, leisure activities, and sex on cognitive decline, to test for a sex modification effect as reported previously (Sienski et al., 2021; Zhang et al., 2021).

We conducted a series of sensitivity analyses for our results. First, we compared baseline characteristics between the included participants and all participants in the 2008 wave of CLHLS to evaluate the representativeness of the 2008 sample. We considered survey-weighted regression to obtain population-representative effect estimates. We adjusted the ordinal logistic regression model by using more informative covariates, such as depressive symptoms and function of activities of daily living. We used linear regression models to check the robustness of the main results by treating cognitive decline as a continuous variable rather than as a categorical variable (models with and without interaction terms).

All probability values were two-tailed, and all CIs were estimated at the 95% level. All analyses were performed using STATA version 16.0 (StataCorp LLC, College Station, TX, USA).

## Results

### Descriptive Findings

**Figure 1:**
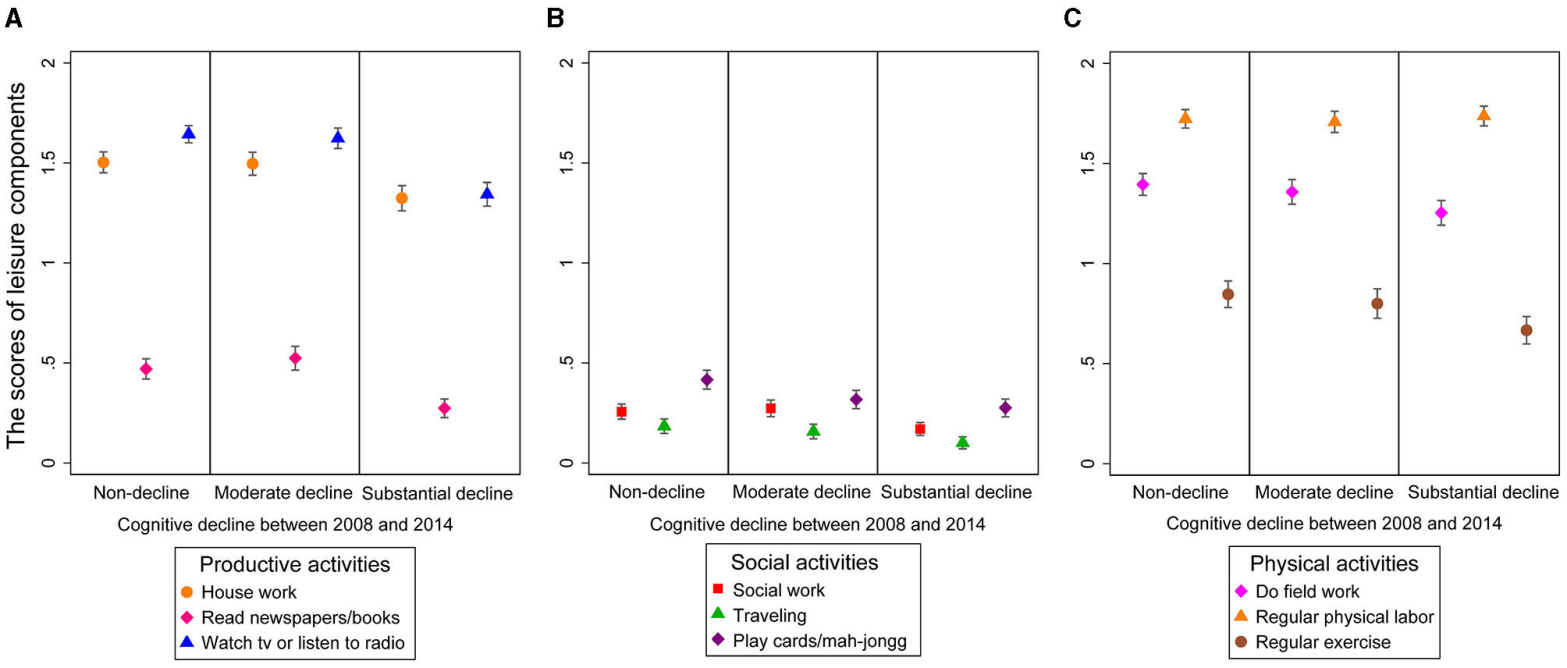
Subtypes leisure [**(A–C)** productive, social, and physical] activities by levels of cognitive decline between 2008 and 2014.

Table 1 summarizes the baseline characteristics of the participants. The 3,017 participants had a mean age of 77.0 (SD: 9.0) with 49.3% female and 588 *APOE* ε4 carriers (19.5%). During the follow-up period from 2008 to 2014, 685 (22.8%) and 723 (23.7%) participants were detected as having moderate and substantial cognitive decline, respectively. Participants who developed substantial cognitive decline have lower ILAs; similar trends were shown regarding productive activities, social activities, and PAs (Figure 1). Participants who were female, were aged, less educated, or had no spouse were more likely to suffer from substantial cognitive decline rather than moderate or nondecline (*P*s < 0.05).

**Table 1:** Characteristics of participants by levels of cognitive decline between 2008 and 2014.

	**Non-decline**	**Moderate decline**	**Substantial decline**	**Total**	***P***
	***n*** **= 1,609**	***n*** **= 685**	***n*** **= 723**	***n*** **= 3,017**	
APOE ε4 allele carrier, *n* (%)	304 (18.89)	127 (18.54)	157 (21.72)	588 (19.49)	0.219
Index of leisure activities, mean, SD	8.30, 2.84	8.26, 2.84	7.14, 2.83	7.95, 2.88	<0.001
Index of productive activities, mean, SD	3.57, 1.34	3.64, 1.41	2.94, 1.48	3.41, 1.43	<0.001
Index of social activities, mean, SD	0.82, 1.19	0.75, 1.08	0.55, 0.98	0.72, 1.11	<0.001
Index of physical activities, mean, SD	3.90, 1.57	3.86, 1.60	3.66, 1.62	3.82, 1.60	0.004
Age, mean, SD	75.81, 8.48	75.58, 8.59	80.86, 9.27	76.96, 8.97	<0.001
**Gender**, ***n*****(%)**					<0.001
Female	755 (46.92)	305 (44.53)	428 (59.20)	1,488 (49.32)	
Male	854 (53.08)	380 (55.47)	295 (40.80)	1,529 (50.68)	
Years of education, mean, SD	3.15, 3.80	3.39, 3.82	1.91, 3.07	2.91, 3.69	<0.001
**Occupation before retirement**, ***n*****(%)**					0.095
White collar	125 (7.77)	60 (8.76)	66 (9.13)	251 (8.32)	
Blue collar	1,339 (83.22)	563 (82.19)	614 (84.92)	2,516 (83.4)	
Others	145 (9.01)	62 (9.05)	43 (5.95)	250 (8.29)	
**Marital status**, ***n*****(%)**					<0.001
Living with a married spouse	665 (41.33)	275 (40.15)	407 (56.29)	1,347 (44.65)	
Have no spouse	944 (58.67)	410 (59.85)	316 (43.71)	1,670 (55.35)	
**Socioeconomic status**, ***n*****(%)**					0.675
Unfavorable	291 (18.09)	116 (16.93)	138 (19.09)	545 (18.06)	
Intermediate	1,078 (67.00)	477 (69.64)	485 (67.08)	2,040 (67.62)	
Favorable	240 (14.92)	92 (13.43)	100 (13.83)	432 (14.32)	
BMI, mean, SD	21.32, 4.39	21.64, 4.29	20.61, 4.46	21.24, 4.41	0.568
Drinking, *n* (%)	408 (25.36)	162 (23.65)	135 (18.67)	705 (23.37)	0.002
Smoking, *n* (%)	447 (27.78)	174 (25.40)	146 (20.19)	767 (25.42)	0.001
DDPI, mean, SD	8.55, 2.25	8.48, 2.25	8.30, 2.28	8.47, 2.27	0.076
Hypertension, *n* (%)	380 (23.59)	164 (23.89)	183 (25.28)	726 (24.06)	0.675
Diabetes, *n* (%)	46 (2.85)	28 (4.13)	23 (3.23)	97 (3.23)	0.289
Heart disease, *n* (%)	147 (9.15)	74 (10.77)	85 (11.81)	307 (10.16)	0.125
Cerebrovascular disease, *n* (%)	83 (5.18)	45 (6.64)	48 (6.59)	176 (5.85)	0.251

*SD, standard deviation; BMI, body mass index; DDPI, diversity of dietary protein intake; APOE ε4, ε4 allele of the apolipoprotein E*.

### Two-Way Interactions: APOE ε4 and Leisure Activities

Table 2 reports the independent associations of *APOE* ε4, ILAs, and the three subtypes of leisure activities (productive activities, social activities, and PAs) with cognitive decline in the cohort. In the fully adjusted model, we observed a significantly higher odds of cognitive decline for individuals possessing *APOE* ε4 alleles (odds ratio, OR = 1.25, 95% CI: 1.03, 1.52; *P* = 0.026). We found that the ILAs (OR = 0.93, 95% CI: 0.89, 0.97; *P* < 0.001), as well as productive activity (OR = 0.90, 95% CI: 0.85, 0.96; *P* = 0.001), and social activity (OR = 0.92, 95% CI: 0.85, 0.99; *P* = 0.026) were significantly associated with lower odds of cognitive decline, respectively. The association between PA and cognitive decline narrowly missed the margin of significance in the fully adjusted model (OR = 0.96, 95% CI: 0.91, 1.01; *P* = 0.083). We did not observe a significant association between *APOE* ε2 allele carriers (ε2/ε2, ε2/ε3) and slower cognitive decline compared with the ε3/ε3 reference (data not shown).

**Table 2:** Association of APOE ε4, Index of leisure activities (ILA), and subtypes of leisure activity with a cognitive decline between 2008 and 2014.

	**Model 1**		**Model 2**		**Model 3**		**Model 4**	
	**OR**	***P***	**OR**	***P***	**OR**	***P***	**OR**	***P***
APOE ε4 (Carrier vs. Noncarrier)	1.25 (1.03, 1.51)	0.023	1.24 (1.03, 1.50)	0.027	1.23 (1.02, 1.50)	0.034	1.25 (1.03, 1.52)	0.026
Index of leisure activities	0.90 (0.88, 0.93)	<0.001	0.93 (0.89, 0.96)	<0.001	0.93 (0.90, 0.97)	<0.001	0.93 (0.89, 0.97)	<0.001
Index of productive activity	0.80 (0.76, 0.85)	<0.001	0.89 (0.85, 0.95)	<0.001	0.91 (0.86, 0.96)	0.002	0.90 (0.85, 0.96)	0.001
Index of social activity	0.84 (0.79, 0.90)	<0.001	0.92 (0.84, 0.97)	0.004	0.92 (0.85, 0.99)	0.026	0.92 (0.85, 0.99)	0.039
Index of physical activity	0.93 (0.89, 0.98)	0.002	0.96 (0.91, 1.00)	0.079	0.95 (0.91, 1.00)	0.050	0.96 (0.91, 1.01)	0.083

*The results derived from the four models were presented as odds ratios with 95% CI. Model 1, unadjusted model. Model 2 adjusted for gender, age, and body mass index. Model 3 adjusted for education, occupation before retirement, marital status, smoking, alcohol drinking, and diversity of dietary protein intake based on Model 2. Model 4 further adjusted for hypertension, diabetes, heart disease, and cerebrovascular disease based on Model 3. APOE ε4, ε4 allele of the apolipoprotein E*.

Table 3 shows the results of two-way interaction analysis of *APOE* ε4 carriage with the dichotomous variable of leisure activities and the three subtypes of leisure activities with cognitive decline. We observed that the interaction terms were principally associated with lower odds of cognitive decline in the fully adjusted model. The interactions of *APOE* ε4 with leisure activities (OR = 0.66, 95% CI: 0.44, 0.97; *P* = 0.018) and with productive activities (OR = 0.65, 95% CI: 0.46, 0.96; *P* = 0.015) reached statistical significance, while the interaction terms were not statistically significant for social activities (OR = 0.73, 95% CI: 0.50, 1.05; *P* = 0.109) or PAs (OR = 0.78, 95% CI: 0.50, 1.21; *P* = 0.241).

**Table 3:** Two-way interaction of APOE ε4, leisure activities, and subtypes of leisure activity on the cognitive decline between 2008 and 2014.

	**Odds ratio**	**95% CI**		***P***
*APOE* ε4	1.58	(1.16, 2.17)	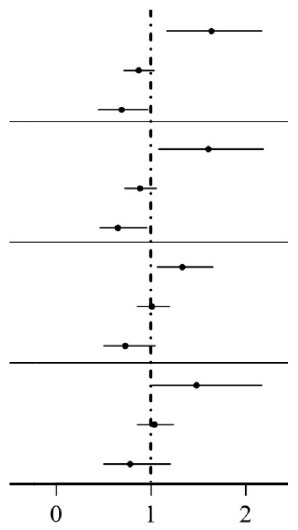	0.004
Leisure activities	0.86	(0.71, 1.04)		0.124
*APOE* ε4 × Leisure activities	0.66	(0.44, 0.97)		0.018
*APOE* ε4	1.55	(1.08, 2.19)		0.015
Productive activities	0.87	(0.72, 1.06)		0.087
*APOE* ε4 × Productive activities	0.65	(0.46, 0.96)		0.015
*APOE* ε4	1.33	(1.06, 1.66)		0.012
Social activities	1.01	(0.85, 1.20)		0.867
*APOE* ε4 × Social activities	0.73	(0.50, 1.05)		0.109
*APOE* ε4	1.48	(1.01, 2.17)		0.045
Physical activities	1.02	(0.85, 1.24)		0.813
*APOE* ε4 × Physical activities	0.78	(0.50, 1.21)		0.241

*APOE ε4: Carrier vs. noncarrier. Leisure activities: ≥8 vs. ≤ 7 of leisure activities index. Productive activities: ≥3 vs. ≤ 2 of productive activities index. Social activities: ≥1 vs. =0 of social activities index. Physical activities (PAs): ≥3 vs. ≤ 2 of physical activities index*.

*The results derived from the full-adjusted models were presented as odds ratios with 95% confidential intervals. The model adjusted for gender age, body mass index, education, occupation before retirement, marital status, smoking, alcohol drinking, diversity of dietary protein intake, hypertension, diabetes, heart disease, and cerebrovascular disease. APOE ε4, ε4 allele of the apolipoprotein E*.

**Figure 2:**
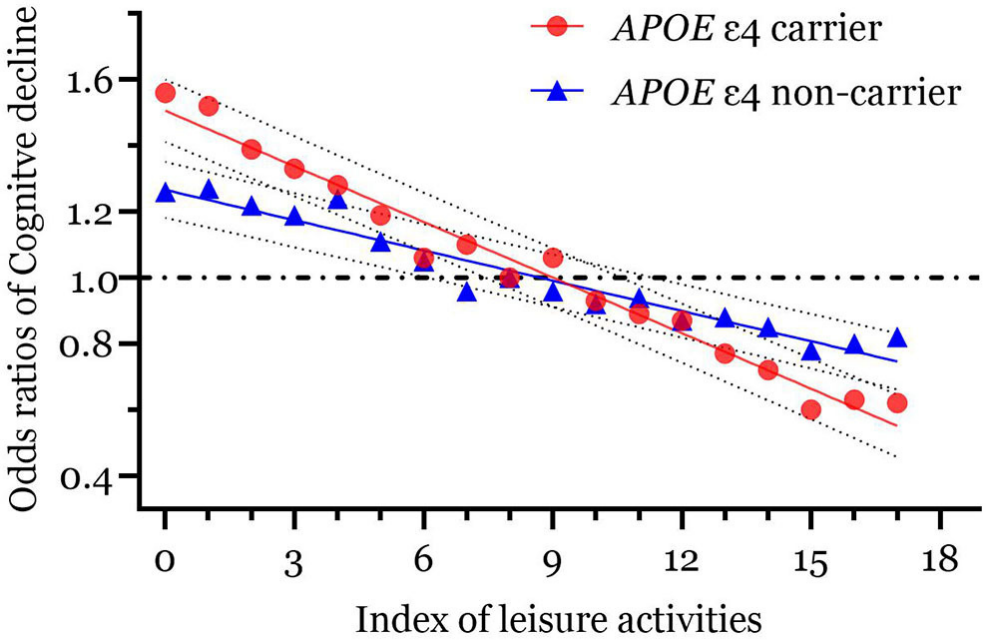
The associations between Index of leisure activities (ILAs) and cognitive decline by the status of possessing *APOE* ε4 allele. *APOE* ε4, ε4 allele of the apolipoprotein E.

We depicted the associations between the ILAs and the OR of cognitive decline stratified by possession of an *APOE* ε4 allele (Figure 2). The association between leisure activities and cognitive decline was more pronounced in the group that possessed *APOE* ε4 alleles in contrast to *APOE* ε4 allele noncarriers (Figure 2).

### Three-Way Interactions: APOE ε4, Sex, and Leisure Activities

The three-way interaction between *APOE* ε4, sex, and leisure activities was not significant (*P*_3−wayinteraction_ = 0.527). Supplementary Figure 2 shows that the interaction is principally driven by female participants having a more marked association between leisure activities and cognitive decline, compared with men.

### Sensitivity Analyses

We compared baseline characteristics between the included participants (3,017) and all participants (Raber et al., 2004; 972) genotyped in the 2008 wave of CLHLS; all comparisons were nonsignificant except for age (Supplementary Table 1). The participants in this study are younger than the cohort of all participants in the 2008 wave of CLHLS (77.9 ± 9.0 vs. 80.6±11.3; *P* < 0.001). In the sensitivity analyses, the associations of *APOE* ε4, ILAs, and three subtypes of leisure activities with cognitive decline were generally consistent with the main findings reported, after we included depressive symptoms, the function of activities of daily living, and population weights (Supplementary Table 2), dropping from the sample individuals who had moderate or severe cognitive impairment at baseline (Supplementary Table 3). We also performed linear regression analysis measuring cognitive decline as a continuous variable (Supplementary Table 4). We observed consistent results for the interaction of *APOE* ε4 with leisure activities and with the three leisure components in the linear regression analysis (Supplementary Table 5), compared with the ordinal logistic regression models in Table 3.

## Discussion

In this study, we found that engagement in leisure activities and *APOE* ε4 carriage had significant and opposite effects on cognitive health, consistent with previous studies. Among the three sub-types of leisure activities, our findings showed that the negative associations with cognitive decline were more pronounced in productive and social activities than in PAs. Our study was one of only a few that reported significant interactions between leisure activities and *APOE* ε*4* on cognitive decline, indicating that practicing leisure activities, particularly productive and social activities, might partly offset the risk of *APOE* ε4-related cognitive decline.

There was consistent epidemiological evidence linking lack of participation in leisure activities with the development of dementia and decline of cognitive function (Wilson et al., 2007; Niti et al., 2008; Akbaraly et al., 2009). A mechanism hypothesized to underlie this linkage was that leisure activities exert protective effects on cognitive function by reducing stress (McEwen, 2002). Stress could cause corticosterone hypersecretion, which downregulates the hippocampal corticosteroid receptors, which in turn dampens the feedback inhibition of the adrenocortical axis leading to further hypersecretion, which eventually harms the hippocampal neurons (Sapolsky et al., 1986). Leisure activities could lead to positive emotional states, such as self-esteem, social competence, and adequate mood, all of which are buffers to stress (Fratiglioni et al., 2004). Findings from the current study provided evidence that higher scores of ILAs were associated with lower odds of cognitive decline, suggesting that integrated leisure activities could protect against cognitive decline.

Results from a sub-group analysis of productive activities, social activities, and PAs showed that among the three sub-types, productive activities have the most robust association with lower odds of cognitive decline, compared with social activities and PAs. Previous studies have shown that productive activities were associated with the reduced risk of dementia and cognitive decline. For example, in a French cohort, knitting, doing odd jobs, gardening, and traveling reduced the risk of dementia (Fabrigoule et al., 1995). In the Nun Study, low density of ideas and low levels of grammatical complexity in autobiographies written in early life were associated with low cognitive test scores in later life (Snowdon et al., 1996). In the Bronx Aging study, reading, playing board games, playing musical instruments, and dancing were associated with a lower risk of dementia (Scarmeas, 2003). It has been suggested that mental stimulation could selectively increase synaptogenesis (Churchill et al., 2002) while accumulating evidence concerning brain plasticity in adult life has shown the existence of angiogenesis, synaptogenesis, and neurogenesis (Fillit et al., 2002). Productive activity, which was more relevant and directly associated with mental stimulation in contrast to social activities and PAs, may create more dendritic branches and synaptic connections and alleviate the amyloid burden in the brain, which improves or preserves cognitive function (Fratiglioni et al., 2004). This was supported by evidence from studies showing that activities with higher cognitive demand have a more robust association with cognition (Hultsch et al., 1999) and may protect against cognitive decline (Katzman, 1995; Wang et al., 2006). Meanwhile, our findings also reinforced the arguments that social activities may affect cognitive health through common pathways by receiving emotional support from relatives and friends thus reducing depression and the adverse effects of stress and associated elevation of cortisol (Bassuk et al., 1999). Previous studies suggested that physical training may promote cerebral blood flow and angiogenesis, decreasing the accumulation of radical oxidative proteins, thus benefitting cognitive function (Kivipelto et al., 2001). We were unable to identify a significant beneficial effect of PA. Notably, the PAs among older people in this study were self-reported in contrast to formal, physical training. It is possible that the lack of professional advice and supervision for the PAs leads to limited power to protect against cognitive decline.

We did not detect the significant association between carrying *APOE* ε2 allele and slower cognitive decline, partly due to the small toward nonneuroprotective effect of *APOE* ε2 at an advanced age. In this study, we reported a significant association between *APOE* ε4 carriage and accelerated cognitive decline, consistent with the established literature. It is reported that *APOE* ε4 risk allele carriers have worse brain profiles, i.e., smaller brain volumes and poorer cognition, than noncarriers (Agosta et al., 2009; Honea et al., 2009; Schuff et al., 2009; Hafsteinsdottir et al., 2012). One study investigated the association of the *APOE* ε4 allele and leisure activity with brain tissue volume, in a population-based cohort of 4,303 non-demented individuals, aged 66–96 years. This study reported that *APOE* ε4 carriers had greater white matter hyperintensities and cerebrospinal fluid volumes than noncarriers but smaller gray matter volumes (Hafsteinsdottir et al., 2012). Individuals with lower leisure activity levels had greater white matter hyperintensities and cerebrospinal fluid volumes, smaller total brain parenchymal, white matter, and gray matter volumes than those with the highest levels of participation (Hafsteinsdottir et al., 2012). Therefore, the study concluded that carriers of the *APOE* ε4 risk allele have worse brain profiles than noncarriers. Participation in leisure activity is a factor associated with brain tissue volume and white matter hyperintensities load and, in turn, affects age-related cognitive decline (Hafsteinsdottir et al., 2012).

Significant interactions were found between leisure activities and *APOE* ε4 carriage in the current study, notably in the sub-type of productive activities. The protective effect of leisure activities on cognitive decline was more pronounced in *APOE* ε4 carriers. Based on the interpretation of brain tissue volume differences between *APOE* ε4 carriers and noncarriers, it has been postulated that individuals carrying the *APOE* ε4 allele may have less effective neural protection and repair mechanisms and maybe thus more dependent on lifestyle-related factors to protect them against cognitive decline and dementia (Mahley and Rall, 2000). Meanwhile, leisure activities, especially productive activities, could reduce rates of hippocampal atrophy (Valenzuela et al., 2008). Therefore, the protective effect of leisure activities, especially productive activities on cognitive decline, would be more pronounced in *APOE* ε4 carriers. Social activities could affect cognitive function by common pathways of reducing stress, and indeed there was a trend toward statistical significance regarding the interaction between social activities and *APOE* ε4 (*P* = 0.109), with the protective effect on cognitive decline more pronounced in *APOE* ε4 carriers. However, a significant interaction between PAs and *APOE* ε4 was not observed. Although some studies suggested that PA may modify the impact of *APOE* ε4 on Alzheimer's disease (AD) and cardiovascular disease (CVD) risk (Bos et al., 2018), however, the evidence was inconclusive; other longitudinal studies found no evidence for an interaction effect between PA and APOE ε4 in cognitive decline in older adults (Folley et al., 2019; Stringa et al., 2020) or found the association of PA with better cognitive function only in among *APOE* ε4 noncarriers (Fernández-Matarrubia et al., 2021). In the current study, our results were in accordance with no interaction. This may suggest that physical stimulations are less pronounced in *APOE* ε4 carriers than mental stimulations, nevertheless, the validity of self-reported physical exercise among older people in China should also be taken as a plausible explanation.

We also found that women were more sensitive to APOE ε4-by-leisure interaction (ILA) in contrast to men. Although the *P*-value of the three-way interaction was not significant, a plot of the interaction between *APOE* ε4 and ILA on cognitive decline showed that this interaction was principally driven by female participants. Established studies showed that lipid metabolism in women is likely to be more affected by APOE genotype than in men (Ungar et al., 2014; Sienski et al., 2021; Zhang et al., 2021). Meanwhile, studies of the hippocampus as a target of stress and sex hormones have revealed a considerable degree of structural plasticity and remodeling in the adult brain that differs between the sexes, with effects more marked in women (McEwen, 2002). In our study, changes in cognitive decline with aging, female *APOE* ε4 carriers were found to be the most sensitive, in contrast with female *APOE* ε4 noncarriers, male *APOE* ε4 carriers, and male *APOE* ε4 noncarriers.

Some aspects of the measurement of leisure activities and interpretation of the results require discussion. First, the activities in the sub-types may not be mutually exclusive. For example, doing housework was assigned to productive activities, but it may also be physically demanding. One of our criteria for separating leisure activities into sub-types was definition in established studies. Activities involved with more brain function would be assigned to productive activities, activities involved with other people would be assigned to social activities, and activities involved with more physical movements would be assigned to PAs. Second, self-reported leisure activities are context-specific due to social environment and culture, therefore, in this study, physical exercise among older people in China could differ from standard physical training in some developed countries. The differentiation of physical labor from leisure activities among older people in China is complex. Since there were still a large proportion of older people living in or having lived in rural areas, they often engaged in fieldwork, such as gardening for leisure, although they may no longer make their living in agriculture. On the other hand, physical exercises for some older people include physically involved activities that do not require specialized guidance, such as square dancing or walking. In those circumstances, the stimulation effect of physical exercise may be disused or not as effective as standard physical training.

Our study findings are of help to better define the target populations for future interventions and to better delineate preventive and therapeutic lifestyle strategies. The identification of lifestyle-gene interactions offers us an opportunity to develop leisure interventions, particularly productive and social activities that will mitigate the detrimental effects of genetic factors including *APOE* ε4 allele carriage. Leisure activities that stimulate cognitive function may be recommended as an intervention to modify the rate of age-associated cognitive decline in older people.

The main strengths of this study are the longitudinal design with a relatively long duration of follow-up (≥6 years) and the use of a nationwide sample of Chinese community-dwelling older adults (*n* = 3,017, with 588 *APOE* ε4 carriers). Another strength was that we classified leisure activities into productive leisure activities, social activities, and PAs for a better interpretation of the effect on cognitive decline. We also conducted a series of sensitive analyses to evaluate the statistical robustness of the results.

Our study has several limitations. First, our study could not distinguish potential reverse causation. This is because we could not “set” leisure activities but only observe them from the survey, therefore, the assumption that participation in leisure activities has a directional effect on cognitive decline could be reversed, e.g., cognitive decline and disease reduce the likelihood of participation in leisure activities. We controlled for potential confounding variables, such as diseases, which were risk factors for cognitive decline so that the association between baseline leisure activities and cognitive decline was not confounded by these factors. Second, we were unable to include more leisure components, such as listening to music, art creation, or writing due to the limitation of the questionnaire design of CLHLS. Third, we did not have a precise physician-based clinical diagnosis of cognitive decline. Therefore, future studies with more sophisticated designs and clinical endpoints are warranted to decipher the mechanisms of gene-by-lifestyle interactions between *APOE* ε4 and leisure activities.

## Conclusion

Our study provided evidence that leisure activities, especially productive and social activities, can potentially offset the detrimental effect of *APOE* ε4 allele carriage on cognitive health. Further research with a clearer definition of the types and intensities of leisure activities is needed for a more precise understanding of this relationship. If substantiated, personalized lifestyle recommendations of having more productive leisure activities for the older people with the *APOE* ε4 allele may bring substantial benefits for maintenance of cognitive health.

## Data Availability Statement

The data analyzed in this study is subject to the following licenses/restrictions: The datasets during and/or analyzed during the current study available from the corresponding author on reasonable request. Requests to access these datasets should be directed to Yao Yao (yaoyao@nsd.pku.edu.cn).

## Author Contributions

YunZ, YY, and YiZ designed the research and directed its implication. YunZ, SF, and YY prepared and analyzed the data and drafted the manuscript. All co-authors revised the manuscript together.

## Funding

This work was supported by the National Natural Science Foundation of China (81900357, 81903392, 81941021, and 72061137004), National Key R&D Program of China (2018YFC2000400), National Social Sciences Foundation of China (19CRK005), China Postdoctoral Science Foundation funded project (2019M663338 and 2019M650359), the U.S. National Institute of Aging of National Institute of Health (P01AG031719), the Duke/Duke-NUS Collaboration Pilot Project, and the Data for Better Health Project of Peking University-Master Kong.

## Conflict of Interest

The authors declare that the research was conducted in the absence of any commercial or financial relationships that could be construed as a potential conflict of interest.

## Publisher's Note

All claims expressed in this article are solely those of the authors and do not necessarily represent those of their affiliated organizations, or those of the publisher, the editors and the reviewers. Any product that may be evaluated in this article, or claim that may be made by its manufacturer, is not guaranteed or endorsed by the publisher.

## Abbreviations

*APOE* ε4: ε4 allele of the apolipoprotein E
CLHLS: Chinese Longitudinal Healthy Longevity Survey
MMSE: Mini-Mental State Examination
SD: standard deviation
CI: confidence interval.
