# Supplementary material for: Leisure Activities, APOE ε4, and Cognitive Decline: A Longitudinal Cohort Study
Source: Front Aging Neurosci. 2021 Sep 20;13:736201. doi: 10.3389/fnagi.2021.736201 (PMC8488387; doi:10.3389/fnagi.2021.736201)
Supplement: Supplementary file 1 [file Presentation_1.pdf]

Supplementary files

Leisure activities, APOE ε4, and cognitive decline: a longitudinal cohort study

Yun Zhang[1,a], Shihui Fu[2,a], Ding Ding[3], Michael W. Lutz [4], Yi Zeng[5,6,*] and Yao Yao[5,6,7,*]

1. School of Sociology and Anthropology, Sun Yat-sen University, Guangzhou, Guangdong 510275, China.
2. Department of Cardiology, Hainan Hospital of Chinese People's Liberation Army General Hospital, Sanya 572000, China.
3. Institute of Neurology, Huashan Hospital, Fudan University, Shanghai, China.
4. Department of Neurology, Duke University School of Medicine, Durham, NC 27710, United States.
5. China Center for Health Development Studies andCenter for Healthy Aging and Development Studies, National School of Development, Peking University, Beijing 100871, China.
6. Center for the Study of Aging and Human Development and Geriatrics Division, Medical School of Duke University, Durham, NC 27710, United States.
7. China Center for Health Development Studies, Peking University, Beijing, China.

Figure S1. Study profile.

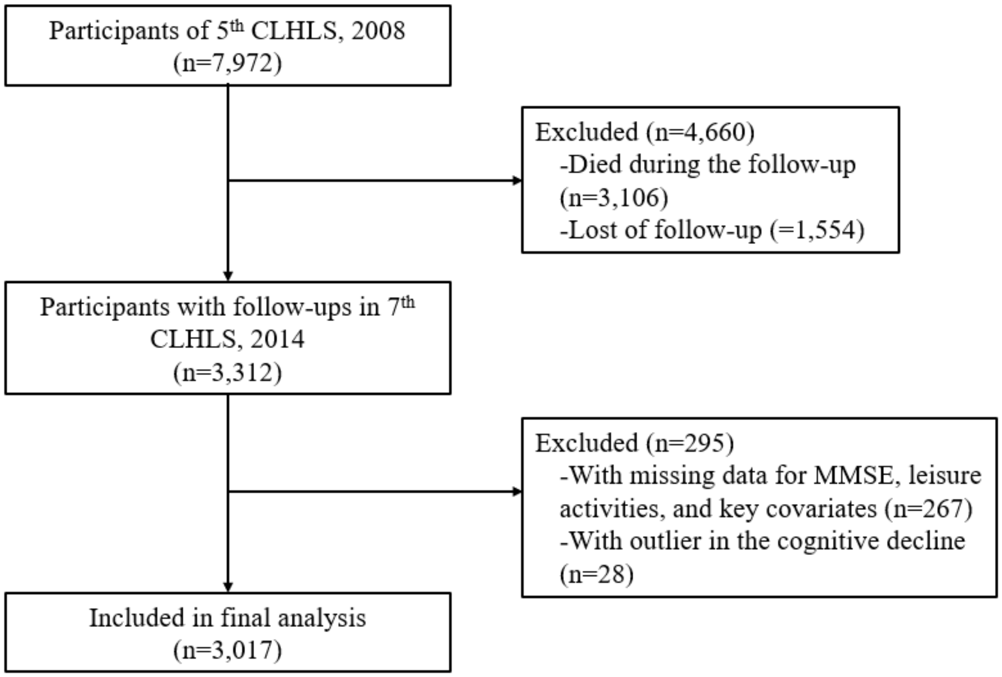

Figure S2. Three-way interactions of APOE ε4, sex, and leisure activities on cognitive decline.

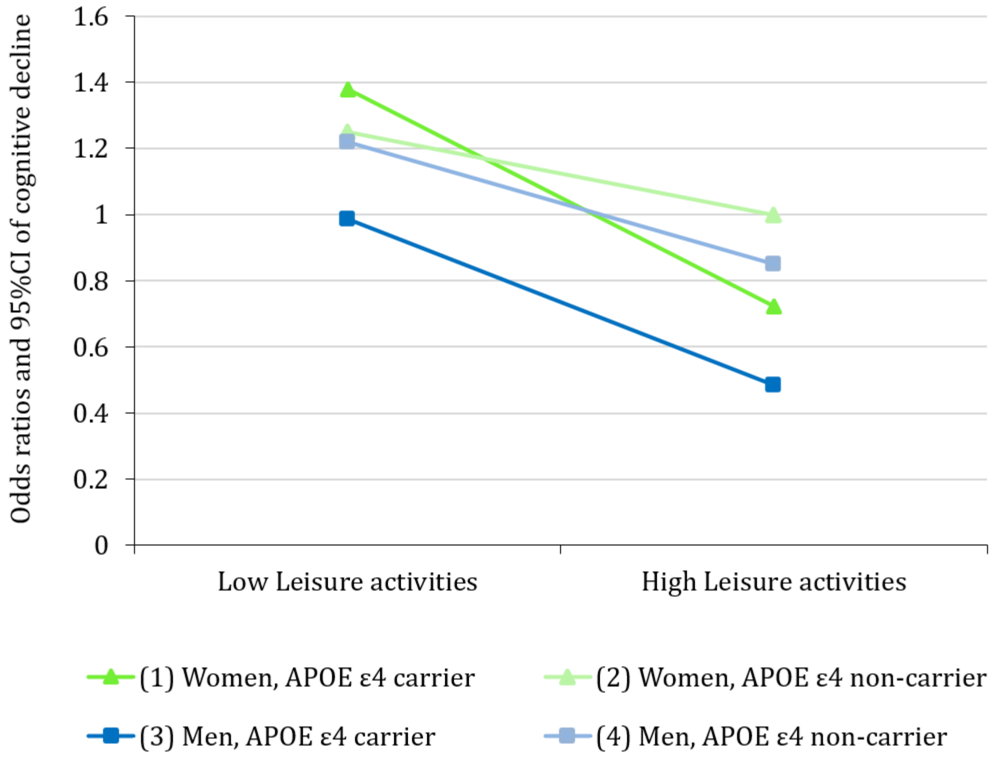

Table S1. Comparison of baseline characteristics between the included participants 3017 and all participants survey and genotyped in the 2008 wave of CLHLS.

	Participants in this study (n=3017)	Participants genotyped in the 2008 wave of CLHLS (n=7972)	*P*
APOE ε4 allele carrier, %	19.49	19.86	0.864
Age, mean, SD	77.0, 9.0	82.6, 11.3	< 0.001
Gender, %			0.671
Female	49.32	48.70	
Male	50.68	51.30	
Years of education, mean, SD	2.91, 3.69	2.54, 3.70	0.294
Occupation before retirement, %			0.413
White collar	8.32	10.35	
Blue collar	83.4	81.32	
others	8.29	8.33	
Marital status, %			0.128
Living with a married spouse	44.65	41.24	
Have no spouse	55.35	58.76	
Socioeconomic status, %			0.677
Unfavorable	18.06	18.87	
Intermediate	67.62	67.02	
Favorable	14.32	14.11	

Table S2. Sensitivity analysis of association of APOE ε4, Index of leisure activities, and subtypes of leisure activity with cognitive decline, considering depressive symptoms, function of activities of daily living, and population weights as covariates.

	Model 4		Model 5	
	OR	*P*	OR	*P*
APOE ε4 (Carrier vs. Non-carrier)	1.25 (1.03, 1.52)	0.026	1.23 (1.02, 1.50)	0.030
Index of leisure activities	0.93 (0.89, 0.97)	<0.001	0.93 (0.90, 0.97)	<0.001
Index of productive activity	0.90 (0.85, 0.96)	0.001	0.90 (0.87, 0.97)	0.004
Index of social activity	0.92 (0.85, 0.99)	0.039	0.91 (0.85, 0.97)	0.012
Index of physical activity	0.96 (0.91, 1.01)	0.083	0.97 (0.90, 1.05)	0.117

Note: The results derived from the four models were presented as odds ratio with 95% confidential intervals. Model 4 adjusted for gender, age, body mass index, education, occupation before retirement, marital status, smoking, alcohol drinking, diversity of dietary protein intake, hypertension, diabetes, heart disease, and cerebrovascular disease. Model 5 additionally adjusted depressive symptoms, function of activities of daily living, and population weights based on Model 4.

Table S3. Sensitivity analysis of association of *APOE* ε4, Index of leisure activities, and subtypes of leisure activity with cognitive decline, excluding the sample who had moderate or severe cognitive impairment at baseline.

	Model 4		Model 5	
	OR	*P*	OR	*P*
APOE ε4 (Carrier vs. Non-carrier)	1.26 (1.03, 1.56)	0.006	1.26 (1.03, 1.52)	0.014
Index of leisure activities	0.92 (0.89, 0.97)	<0.001	0.94 (0.89, 0.98)	0.008
Index of productive activity	0.90 (0.85, 0.96)	<0.001	0.90 (0.84, 0.96)	<0.001
Index of social activity	0.91 (0.85, 1.00)	0.049	0.92 (0.85, 0.99)	0.039
Index of physical activity	0.95 (0.91, 1.04)	0.104	0.96 (0.91, 1.01)	0.083

Note: The results derived from the four models were presented as odds ratio with 95% confidential intervals. Model 4 adjusted for gender, age, body mass index, education, occupation before retirement, marital status, smoking, alcohol drinking, diversity of dietary protein intake, hypertension, diabetes, heart disease, and cerebrovascular disease. Model 5 additionally adjusted depressive symptoms, function of activities of daily living, and population weights based on Model 4.

Table S4. Sensitivity analysis of association of *APOE* ε4, Index of leisure activities, and subtypes of leisure activity with cognitive decline (as continuous variables).

	Model 4		Model 5	
	β	*P*	β	*P*
APOE ε4 (Carrier vs. Non-carrier)	-0.726	0.007	-0.786	0.014
Index of leisure activities	0.295	<0.001	0.290	<0.001
Index of productive activity	0.395	<0.001	0.382	<0.001
Index of social activity	0.383	<0.001	0.379	<0.001
Index of physical activity	0.138	0.041	0.124	0.076

Note: The results derived from the four models were presented as odds ratio with 95% confidential intervals. Model 4 adjusted for gender, age, body mass index, education, occupation before retirement, marital status, smoking, alcohol drinking, diversity of dietary protein intake, hypertension, diabetes, heart disease, and cerebrovascular disease. Model 5 additionally adjusted depressive symptoms, function of activities of daily living, and population weights based on Model 4.

Table S5. Sensitivity analysis of two-way interaction of *APOE* ε4, leisure activities, and subtypes of leisure activity on cognitive decline (as continuous variable) between 2008 and 2014.

	Model 1	Model 2	Model 4
APOE ε4	-1.08 (-1.92, -0.24) **	-1.23 (-2.04, -0.41) ***	-1.29 (-2.12, -0.46) ***
Leisure activities	1.44 (0.97. 1.91) ***	0.54 (0.06, 1.02)**	0.49 (-0.02, 0.99) *
APOE ε4 ×Leisure activities	0.96 (-0.10, 2.04) *	1.06 (0.01, 2.11) **	1.28 (0.21, 2.35) **
APOE ε4	-1.27 (-2.30, -0.24) **	-1.56 (-2.56, -0.56) ***	-1.57 (-2.58, -0.55) ***
Productive activities	1.46 (0.94. 1.98) ***	0.44 (-0.09, 0.97)	0.38 (-0.17, 0.93)
APOE ε4 ×Productive activities	1.01 (-0.18, 2.21) *	1.30 (0.13, 2.47) **	1.42 (0.23, 2.60) **
APOE ε4	-0.96 (-1.64, -0.28) ***	-1.07 (-1.73, -0.42) ***	-1.04 (-1.7,1 -0.37) ***
Social activities	0.62 (0.14. 1.10) **	0.09 (-0.38, 0.56)	0.08 (-0.41, 0.58)
APOE ε4 ×Social activities	1.16 (0.08, 2.24) **	1.24 (0.15, 2.29) **	1.30 (0.23, 2.37) **
APOE ε4	-0.88 (-1.94, 0.19)	-1.09 (-2.11, -0.05) **	-0.83 (-1.89, 0.23)
Physical activities	0.07 (-0.46. 0.59)	-0.23 (-0.75, 0.27)	-0.25 (-0.77, 0.24)
APOE ε4 ×Physical activities	0.50 (-0.73, 1.73)	0.62 (-0.53, 1.84)	0.41 (-0.81, 1.63)

Note: APOE ε4: Carrier vs. non-carrier. Leisure activities: >=8 vs. <=7 of leisure activities index. Productive activities: >=3 vs. <=2 of productive activities index. Social activities: >=1 vs. =0 of social activities index. Physical activities: >=3 vs. <=2 of productive activities index.

The results derived from the four models were presented as odds ratio with 95% confidential intervals. Model 1, unadjusted model. Model 2 adjusted for gender age, and body mass index. Model 4 further adjusted for education, occupation before retirement, marital status, smoking, alcohol drinking, diversity of dietary protein intake, hypertension, diabetes, heart disease, and cerebrovascular disease.

$^{*}P < 0.1$, $^{**}P < 0.05$, $^{***}P < 0.01$.
